# Automated Behavioral Text Messaging and Face-to-Face Intervention for Parents of Overweight or Obese Preschool Children: Results From a Pilot Study

**DOI:** 10.2196/mhealth.4398

**Published:** 2016-03-14

**Authors:** Lisa Militello, Bernadette Mazurek Melnyk, Eric B Hekler, Leigh Small, Diana Jacobson

**Affiliations:** ^1^ The Ohio State University College of Nursing Columbus, OH United States; ^2^ Arizona State University School of Nutrition and Health Promotion Phoenix, AZ United States; ^3^ Virginia Commonwealth University School of Nursing Richmond, VA United States; ^4^ Arizona State University College of Nursing and Health Innovation Phoenix, AZ United States

**Keywords:** Child, Obesity, Overweight, Health Behavior, Text Messaging/SMS, Mobile Health, Parents, Psychology, Behavior Therapy, Telemedicine

## Abstract

**Background:**

Children are 5 times more likely to be overweight at the age of 12 years if they are overweight during the preschool period.

**Objective:**

The purpose of this study was to establish the feasibility, acceptability, and preliminary effects of a cognitive behavioral intervention (TEXT2COPE) synergized with tailored mobile technology (mHealth) on the healthy lifestyle behaviors of parents of overweight and obese preschoolers delivered in a primary care setting.

**Methods:**

Fifteen preschooler-parent dyads recruited through primary care clinics completed a manualized 7-week cognitive behavioral skills building intervention. Beck’s Cognitive Theory guided the TEXT2COPE intervention content and Fogg’s Behavior Model guided the implementation. The intervention employed a combination of face-to-face clinic visits and ecological momentary interventions using text messaging (short message service, SMS). To enhance the intervention’s relevance to the family’s needs, parents dictated the wording of the text messages and also were able to adapt the frequency and timing of delivery throughout program implementation.

**Results:**

Self-reported findings indicate that the program is feasible and acceptable in this population. The intervention showed preliminary effects with significant improvements on parental knowledge about nutrition (*P*=.001) and physical activity (*P*=.012) for their children, parental beliefs (*P*=.001) toward healthy lifestyles, and parental behaviors (*P*=.040) toward engaging in healthy lifestyle choices for their children. Effect sizes were medium to large for all variables. The timing, frequency, and wording of the text messages were tailored to the individual families, with 69% of parents (9/13) increasing the frequency of the tailored SMS from being sent once weekly to as many as 5 times a week.

**Conclusions:**

Utilizing a cognitive behavioral skills intervention with SMS has great potential for supporting clinical care of overweight and obese preschool children and their families. Further exploration of the potential effects on health and behavioral outcomes is warranted.

## Introduction

### Background

The preschool age group is a priority for surveillance and intense intervention because obesity trends in this group are a predictor of trends in older children and adults [1-3]. Children are 5 times more likely to be overweight at the age of 12 years if they are overweight during the preschool period, and 60% of overweight preschoolers are overweight at the age of 12 years [4]. Yet, challenges arise as parents perceive use of the term obesity to be stigmatizing and blaming [5,6], and a sizable number of pediatricians, pediatric nurse practitioners, and registered dietitians perceive themselves as having low proficiency in behavioral management strategies and parenting management techniques related to obesity [7]. Feasible and acceptable interventions to promote healthy lifestyle choices are greatly needed, particularly for young children prior to the formation of poor lifestyle choices and associated illness.

Evidence suggests that parents desire personalized information relevant to their child and are enthusiastic about receiving text messages (short message service, SMS) endorsed by their child’s primary care provider [8]. mHealth interventions show promise based on evidence using SMS to remind and cue participants to act [8-12]. Interventions using SMS can be successful as reminders about disease management behaviors (ie, medication adherence, blood glucose monitoring) and tailored, interactive, and family-centered interventions can be supplemented with mobile technology to facilitate behavior change [13]. Yet, there are very few studies aimed at promoting behavior change in conjunction with text messaging (SMS) in pediatric populations [13].

Therefore, the purpose of this study was to establish the feasibility, acceptability, and preliminary effects of a 7-session cognitive behavioral intervention combined with tailored and adaptive SMS regarding healthy lifestyle beliefs, perceived difficulty, and behaviors of parents of overweight and obese (OW/OB) preschoolers (aged 3-5 years) delivered in a primary care setting. The first aim was to examine the feasibility and acceptability of the intervention as determined by rates of participant retention, adherence to the study protocol, and response to participant evaluation. The second aim was to evaluate preliminary effects of the intervention on parental self-reported nutrition/physical activity knowledge, healthy lifestyle beliefs, perceived difficulty, and healthy lifestyle behaviors determined by pretest-posttest scores and effect sizes.

### Theoretical Framework

Previous evidence [14,15] suggests that individuals who cognitively appraise healthy lifestyle choices as more difficult are less likely to have intentions to make these choices and engage in healthy lifestyle behaviors. The effects of negative cognitions about oneself are profound when there are skill deficits (eg, poor problem-solving skills, cognitive distortions, and failure to attribute positive outcomes to one’s behavior) [15]. Cognitive behavioral therapy (CBT) emphasizes short-term, problem-focused cognitive and behavioral intervention strategies derived from the science and theory of learning and cognition [16]. Using these guiding principles, the TEXT2COPE intervention was adapted from the COPE/Healthy Lifestyles Thinking, Emotion, Exercise, and Nutrition (TEEN) Intervention Program [15,17-19], and a combination of the Be Beary Healthy [20] and the Parents Lead Active Youth (PLAY) programs [21]. The content manual was created following recommendations for developing print-based tailored interventions [22,23]. Through a 7-session manualized intervention, participants were educated about healthy nutrition, physical activity, and building cognitive and behavioral skills to facilitate healthy lifestyle choices. Cognitive Theory (CT) [24-26] provided the foundation for the selection of study variables (parental knowledge, perceived difficulty, beliefs, and behaviors). With an emphasis on how thinking affects behaviors and emotions, the intervention content comprised problem-solving skills, methods to overcome barriers, goal setting, positive self-talk, and restructuring negative thoughts (Table 1).

**Table:** 

TEXT2COPE Intervention Program Cognitive Behavioral Skills Content	Session Mode
Healthy lifestyles and the thinking, feeling, behaving triangle; basic recommendations for nutrition & physical activity in preschoolers; goal setting	Session 1 Face-to-face AND text messaging
Information on physical activity and nutrition, including appropriate portion sizes, healthy eating, and food groups	Session 2 Self-study AND text messaging
Barriers to goal progression and overcoming barriers through problem solving and cue recognition	Session 3 Face-to-face AND text messaging
Positive thinking and self-talk related to healthy lifestyle behaviors	Session 4 Self-study AND text messaging
Cognitive reframing—with an emphasis on physical and emotional responses to stress and how positive beliefs can help to reframe cognitions and promote positive coping	Session 5 Face-to-face AND text messaging
Effective communication, stress, and coping	Session 6 Self-study AND text messaging

**Figure 1:**
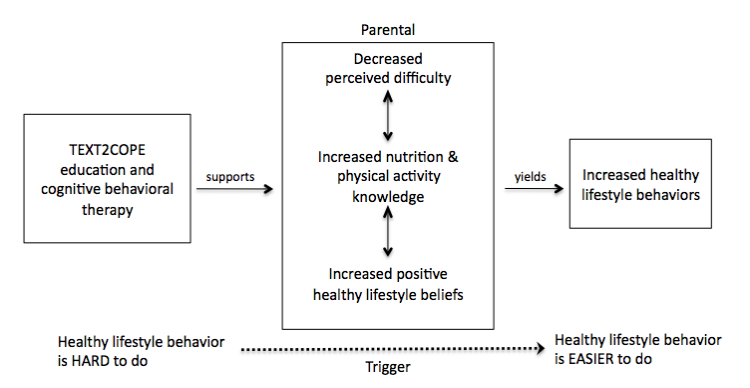
Conceptual model of the TEXT2COPE program.

Aspects of Fogg’s Behavior Model (FBM) informed the overarching strategy for the study [27,28]. In FBM, 3 principal elements (motivation, ability, trigger) are emphasized to better understand the process of behavior change. Triggers serve to facilitate, spark, or signal an action through external or routine stimuli [28]. Triggers that spark, motivate behavior; triggers that facilitate, make a behavior easier; triggers that signal, remind [28]. In this study, a *trigger* was restricted to the purpose of identifying a concept that prompts or calls a person to act. This prompt may be tailored to the target user’s context [27,28]. Text messaging was selected as a tool to trigger skills to promote positive cognitive reframing and subsequent healthy lifestyle behavior choices due to its simplicity, broad user base, and ability to be individualized to the user. Fogg describes triggers as *hot* and *cold* [27]. Hot triggers are presented when users can take action (eg, walking in the kitchen and getting something from the fridge). Cold triggers are presented when users cannot take action at that moment (eg, getting a text message while driving a car). Utilization of hot triggers is more closely associated with a behavioral response [27]. FBM highlights a shift in behavior moving from *hard to do* to *easy to do* [27,28]. The intent of the SMS was to reinforce positive skill enactment and to disrupt repeated negative actions. The blended CT and FBM conceptual model guiding this study is depicted in Figure 1, and offers one approach to healthy lifestyle behavior change for parents of OW/OB preschoolers.

## Methods

### Design and Sample

This pilot study used a one-group pre and posttest preexperimental design approved by the Arizona State University Institutional Review Board. Participants were recruited from 3 pediatric primary care practices. To be included, the primary caretaker (hereafter referred to as the *parent*) of an OW/OB preschooler aged 3 through 5 years had to (a) have a preschooler with a medical diagnosis of OW/OB, defined as a BMI percentile of 85% or above; (b) possess an active mobile phone with text-messaging capability; (c) be between the ages of 18 and 45 years; and (d) give consent for participation.

**Figure 2:**
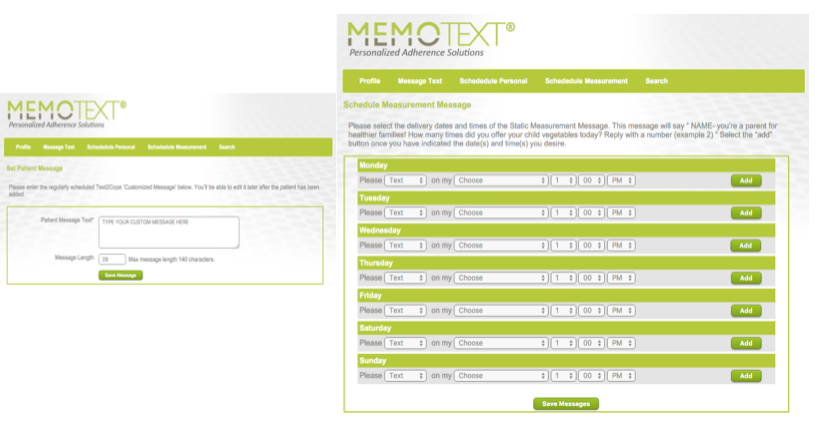
TEXT2COPE-Memotext application

The principal investigator (PI) partnered with MEMOTEXT Corporation to develop software specific to this project for delivering/receiving SMS data. Participant confidentiality was secured through MEMOTEXT’s privacy policy and data were used in accordance with all applicable laws and the Personal Information Protection and Electronic Documents Act. Per the study protocol, no personal health information was communicated via SMS. Both static and tailored messages were entered into the SMS application used for this project. A sample of the software shown in Figure 2 highlights the simplicity of scheduling both a series of tailored and static of messages.

### Recruitment and Enrollment

**Figure 3:**
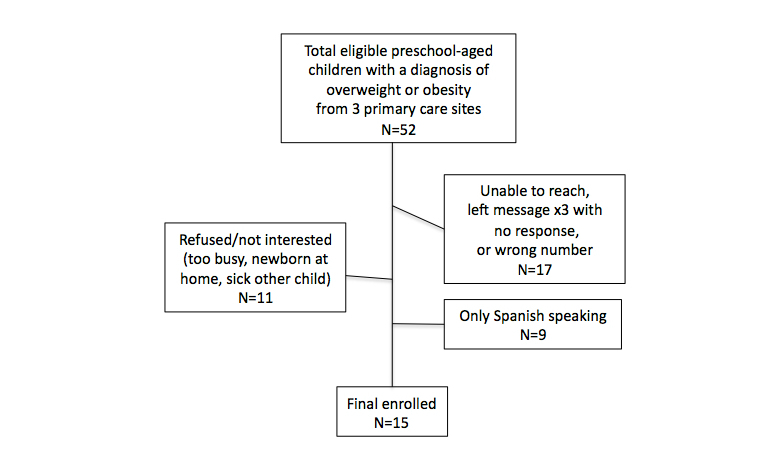
Flow diagram of participant recruitment.

The clinics preferred a combination of burst and serial recruitment efforts. In all of the offices, a support staff member (eg, medical assistant, receptionist) was identified to serve as a recruitment facilitator. The clinic staff member phoned and/or mailed a scripted recruitment letter to all participants identified as having a diagnosis of OW/OB based on provider referral or billing code, and who met eligibility criteria. Recruitment occurred from March to November 2013. Figure 3 depicts participant recruitment. A convenience sample of 15 parent-preschooler dyads (13 parents, 15 preschoolers; 2 families had 2 children who met inclusion criteria) gave consent and were enrolled. This sample size allowed for preliminary analysis. In appreciation of participants’ time and associated costs for text messages, upon completion of baseline instruments, a $10 gift card to a local grocery store was given. Similarly, upon completion of the posttest, a gift card in the amount of $20 was presented.

### Intervention

The intervention implementation consisted of 4 key strategies: (a) face-to-face visits (covering educational information and cognitive behavioral skills), (b) reminders, (c) triggers, and (d) reinforcements. Table 2 details the content covered in each session relevant to the needs and knowledge base of the families. Two families had two children enrolled in the study. However, skills and goals were family-centered versus child-centered, so this did not pose any challenges. Skills and education training occurred during the face-to-face clinic visits. After baseline data were collected, all parents were given a hard copy and an audio compact disc (CD) of the TEXT2COPE manual. The manual served as a reminder of the cognitive behavioral skills and was used to keep the principal investigator (PI) and parents on task. The text messages were designed to trigger the skill or new behavior. Lastly, homework reinforced skills and was reviewed during clinic visits.

**Table 2:** TEXT2COPE intervention implementation.

Session	TEXT2COPE Intervention Program Cognitive Behavioral Skills Content	Mode
1	Healthy lifestyles and the thinking, feeling, behaving triangle; basic recommendations for nutrition & physical activity in preschoolers; goal setting	Face-to-face AND text messaging
2	Information on physical activity and nutrition, including appropriate portion sizes, healthy eating, and food groups	Self-study AND text messaging
3	Barriers to goal progression and overcoming barriers through problem solving and cue recognition	Face-to-face AND text messaging
4	Positive thinking and self-talk related to healthy lifestyle behaviors	Self-study AND text messaging
5	Cognitive reframing—with an emphasis on physical and emotional responses to stress and how positive beliefs can help to reframe cognitions and promote positive coping	Face-to-face AND text messaging
6	Effective communication, stress, and coping	Self-study AND text messaging
7	Putting it all together; integration of knowledge and skills	Face-to-face

### Text Message Support

**Figure 4:**
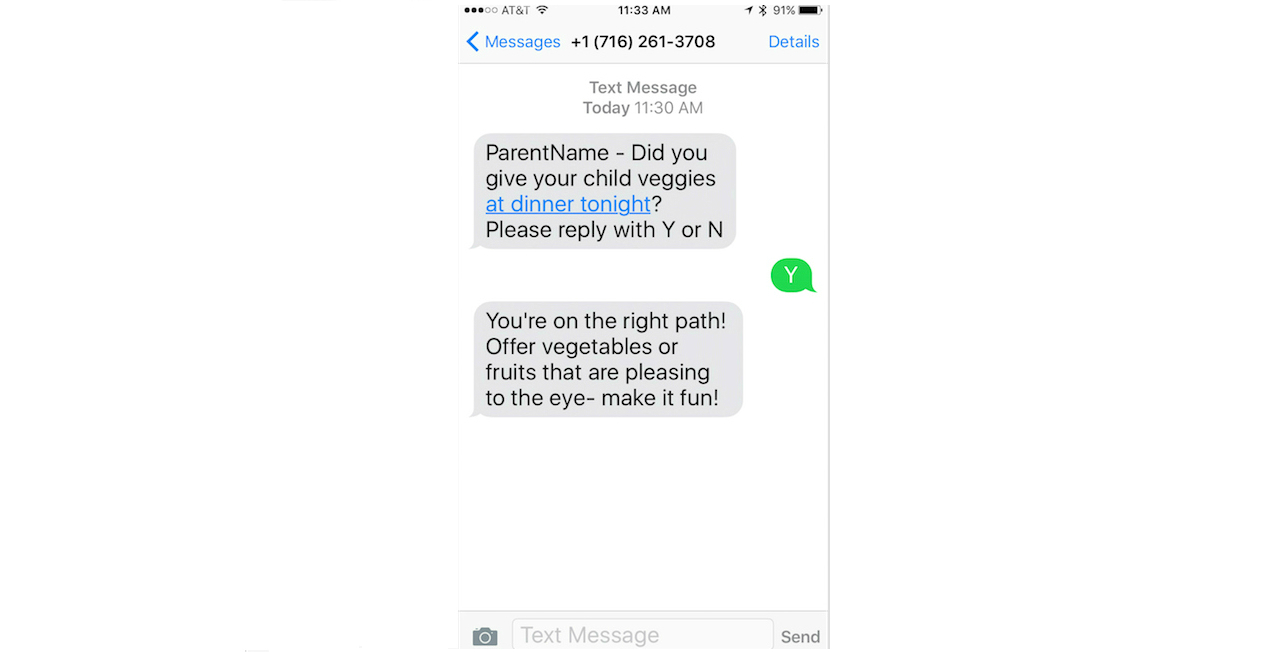
Static text message and support.

Participants responded to static test messages sent twice weekly. For simplicity, because of related health benefits, and evidence indicating that most preschoolers do not eat the recommended daily serving of vegetables, the authors collectively decided to use vegetable consumption as the topic of the static text messages. The use of 2-way weekly text messages (sent/received) is consistent with previous literature [29-34]. Upon sending a response to the static text message, parents received immediate automated SMS feedback generated from a library of text messages developed using *if, then* algorithms. The content of the SMS library of responses was derived from the TEXT2COPE manual used during the face-to-face and self-study components of the intervention. For instance, when the text message “[Parent name] did you give your child veggies at dinner tonight, please reply with Y or N” is sent, an “N” response may elicit the automated skill-building response, “Fear of new foods is common in children—try offering the same vegetable many times in different ways.” Conversely, if the parent responded “Y” to offering veggies, the library would generate a message to reinforce positive behaviors, such as “Be proud of yourself, you’re getting your child off to a healthy start!” Figure 4 illustrates a sample static text message, participant response, and automated support.

To enhance SMS support, parents were taught the difference between hot and cold triggers. At the end of each face-to-face visit, each parent developed a custom text message intended to hot trigger a skill at home. By having participants select the skill to work on, the general verbiage of the prompt, and the day(s) and time each week that they would like to receive the prompt, parents were able to reflect on their own family needs and tailor the intervention content accordingly. The goal was to customize the text message to the families and deliver it at just the right time. Parents were encouraged to create a text message that would “speak to them” regarding that week’s skill or goal. If a parent was unable to articulate a text message, the PI reviewed the session content with the participant until the parent was able to verbalize comprehension and subsequent SMS content. Test messages varied depending on the participant and included the following: the parents’ own words, a mixture of PI and parent words (up to 140 characters), examples from the manual tailored by participants, and a combination of a tip with a page reference from the manual for further information. Examples of the text messages crafted by parents included, “Run, laugh, chase the little guy around. Keep moving!!! Repeat it, BELIEVE IT! You’ve got this!!!!” or “Kick your own butt for 2 minutes! Follow your exercise plan and believe in yourself, now go!” “Wake up sleepy head - you'll regret it if you don't. RUN RUN RUN! you're the Role Model. Don't hit snooze!”

Since the individual ultimately chose the SMS content, each text message took on the tone preferred by the participant (ie, funny or authoritative).

### Manipulation Checks and Fidelity

Homework from the previous session was reviewed at each face-to-face visit. To ensure reception and comprehension, parents were asked to respond to the static text messages. An additional log for each text message sent and received was maintained throughout the study. Two of the authors reviewed all text messages.

A subset of the SMS data reflected a quality control population. Outside of the iterative testing phase of the MEMOTEXT-TEXT2COPE software, the PI included a confederate participant to receive text messages at the same time the MEMOTEXT system was *live*. This was done to explore the system’s delivery and response to potential aberrant text messages from participants. For instance, when one of the tailored text messages was sent, the respondent replied, “Who is this?” This text message was then flagged by the MEMOTEXT staff and then emailed to the PI within 24 hours of being sent. Similarly, for the static text messages, when participant responses occurred outside of *Y* and *N*, the MEMOTEXT staff responded by forwarding an email with the response to the study PI within 24 hours. MEMOTEXT was able to confirm sending text messages. However, unless text message delivery was verified from an aberrant response, delivery was confirmed by self-report at face-to-face visits. This quality control provided information about the logistics built in by the software company to handle such situations and subsequent communication. These features were critical to explore when considering the evidence needed to implement such technology into clinical practice.

### Measures

Measures used in this pilot study assessed parental demographics and child anthropometric measures, parental nutrition and physical activity knowledge as it related to their child [35,36], parental healthy lifestyle beliefs [36,37] and behaviors [36,38] with respect to their child, perceived difficulty towards making healthy lifestyle choices for their child [38], and a parent evaluation form. Table 3 provides a summary of data collection.

**Table 3:** Summary of data collection.

Construct	Instrument	Validity	Reliability^a^	Data Collection
Demographics	Demographic Questionnaire		NA	T1^b^
Parent Evaluation	Exit Interview		NA	T2^c^
Healthy Lifestyles Beliefs Scale	Healthy Lifestyles Beliefs Scale	Content, Construct	.86	T1, T2
Perceived Difficulty	Perceived Difficulty Scale	Content, Construct	.92	T1, T2
Knowledge	Nutrition Knowledge	Content, Construct	.74	T1, T2
Knowledge	Physical Activity Knowledge	Content, Construct	.52	T1, T2
Healthy Lifestyle Behaviors - Parent	Healthy Lifestyle Behaviors Scale	Content, Construct	.78	T1, T2

^a^ Cronbach’s alpha

^b^ T1: Time 1

^c^ T2: Time 2

### Statistical Analysis

All analyses were conducted using SPSS 21. For normally distributed data (nutrition knowledge, perceived difficulty, healthy lifestyle behaviors), paired sample *t* tests were used to evaluate change over time. Effect size measured with Cohen’s *d* for paired samples determined the effect of the intervention. Given the small sample size, two of the outcome variables (activity knowledge, healthy lifestyle beliefs) were nonnormally distributed per the Shapiro-Wilk and Kolmogorov-Smirnov tests. Neither a reflected log_10_ nor a reflected square root transformation corrected the distribution. Therefore, the Wilcoxon signed-rank test for nonparametric data was used to evaluate change over time. The effect size of the Wilcoxon signed-rank test is reported using r code.

## Results

### Demographics

Mothers were predominant study participants and caregivers, consistent with other family-based interventions [14,20,34,39]. Anthropometric data were not obtained for one preschooler who did not attend any face-to-face visits. The mean age of preschool participants was 54.47 months, with a mean BMI of 19.48. Half of the preschoolers met criteria for a diagnosis of overweight (7/14, 50%), and half of the sample met diagnostic criteria for obesity (n=7/14, 50%). Of the 7 children diagnosed with obesity, 6 had BMI percentiles greater than the 99^th^ percentile. Table 4 highlights the demographic data.

**Table 4:** Descriptive characteristics of TEXT2COPE parents and preschoolers.

Participant	Characteristic		n (%)
**Parent** (n=13)	**Age (years)**	18-24	3 (24)
		25-34	5 (38)
		35-44	5 (38)
	**Gender**	Female	13 (100)
		Male	0 (0)
	**Race/Ethnicity**	Caucasian	4 (31)
		African American	1 (8)
		Hispanic/Latino	8 (61)
	**Education**	Less than high school	1 (8)
		General equivalency diploma/high school	5 (38)
		Some college	3 (23)
		4-year college	3 (23)
		Master’s degree	1 (8)
	**Annual Income**	Less than $10,000	5 (38)
		$10,000-$19,000	4 (31)
		$20,000-$29,000	1 (8)
		$40,000-$49,000	1 (8)
		$70,000 or more	2 (15)
	**Public Assistance**	Yes	11 (85)
		No	2 (15)
**Child** (n=15)	**Age**	3 years	2 (13)
		4 years	5 (33)
		5 years	8 (54)
	**Gender**	Female	5 (33)
		Male	10 (67)

### Feasibility, Acceptability, and Preliminary Effects

Parents reported the program to be helpful 100% of the time and 100% of parents indicated that they would recommend TEXT2COPE to other parents of preschoolers. Qualitative feedback included, “The program doesn’t just tell you to be healthy, it shows you how,” and participants noted that it included messages to “Keep trying, don’t give up,” and “Keep pushing towards my goals.” Thematic saturation occurred for goal setting (10/13, 77%) as the most-liked content of the program. No saturation occurred for topics least liked.

Paired *t* tests indicated that parents in the TEXT2COPE program significantly improved scores for nutrition knowledge (95% CI for mean difference -4.33 to -1.54, *P*=.001) and healthy lifestyle behaviors (95% CI for mean difference -9.34 to -0.24, *P*=.04), but not perceived difficulty (95% CI for mean difference -9.56 to 1.87, *P*=.168). Using Wilcoxon signed-rank test for nonparametric data, parental beliefs about their ability to engage in a healthy lifestyle significantly increased from pre to posttest (95% CI for mean difference -16.78 to 1.31, *P*=.001). A Wilcoxon signed-rank test showed significant effects for activity knowledge (*Z*=-2.507, *P*=.012, *R*=0.46) and healthy lifestyle beliefs (*Z*=-3.317, *P*=.001, *R*=.61). Collectively, there were significant gains for 4 out of 5 outcome measures (nutrition knowledge, activity knowledge, healthy lifestyle beliefs, healthy lifestyle behaviors). Effect sizes were medium to large for all study variables.

**Table 5:** Self-reported cognitive and behavioral outcomes for the TEXT2COPE group.

Instrument	Pretest Mean (SD)	Posttest Mean (SD)	Effect Size	*P* Value
Nutrition Knowledge	14.60 (3.46)	17.53 (1.73)	1.07^a^	.001
Activity Knowledge	9.93 (1.58)	11.00 (1.07)	.45^b^	.012
Parental Healthy Beliefs	79.53 (8.10)	83.00 (7.63)	.61^b^	.001
Parental Healthy Behaviors	46.20 (8.37)	51.00 (7.83)	.59^a^	.040
Perceived Difficulty ^c^	38.46 (11.33) ^a^	42.31 (10.94) ^a^	.50^a^	.168

^a^ Effect size for Cohen’s *d* (parametric): .2 small, .5 medium, .8 large

^b^ Effect size for R code (nonparametric): .1 small, .3 medium, .5 large

^c^ Higher scores on the Perceived Difficulty scale indicate less perceived difficulty (easier to do)

### Retention and Attrition

Parent participants were defined as completing the program if all of the content in the 7 TEXT2COPE sessions were covered and parents completed pretest and posttest measures. Findings reflect a 100% retention rate. Although missed or canceled appointments did occur, participants were able to continue the program through provider understanding, rescheduling of appointments, and counseling on the benefits of healthy lifestyle habit establishment relevant to the family’s needs. It is believed that customization of the content relative to the family, routine follow-up in person, and supplemental text messages facilitated patient engagement.

### Program Adherence

The 4 face-to-face visits, which lasted approximately 20-30 minutes to mimic the length of a standard office visit in primary care, were conducted during an outpatient visit at the child’s primary care office. As in other studies [21,39], parents in the TEXT2COPE program attended visits with or without their child; however, they were strongly encouraged to practice and model the skills at home with their child. All participants had to reschedule at least one clinic visit. Reasons for rescheduling included: (a) work schedule, (b) sick child, (c) forgot about the appointment/lost track of time, or (d) lack of transportation. Participants completed the face-to-face visits over the course of 3 to 7 weeks, with 5 being the median number of weeks needed to go through the course content. The PI hosted a *make-up* session lasting 40-60 minutes to cover additional materials.

### Tailored and Adapted Text Messages

A total of 291 text messages were sent to parents: 138 tailored text messages, 105 static text messages, and 53 automated feedback text messages. The number of text messages sent to each participant varied from 7 to 39, with a mean of 22.31 (SD 9.47). Tailored text messages per participant ranged from 3 to 20 (mean 10.62, SD 5.45). The number of static text messages sent to each participant ranged from 4 to 12 (mean 8.00, SD 2.42). Overall, 69% of parents (9/13) changed the frequency of tailored text messages, increasing delivery from once weekly to as many as 5 times a week. Timing (when the message was delivered) was evaluated by dividing the day into 3 segments: morning (6:01 am-12:00 pm), afternoon (12:01 pm-6:00 pm), and evening (6:01 pm and later). Parents favored receiving tailored text messages in the afternoon (58.8% of the time), coinciding with parents’ perceived need for support (and when the message would most likely serve as a hot trigger). Peak timing occurred during the hours when children were home from school, the workday ended, and dinner/afterschool activities coincided. All parents reported SMS frequency to be “just right,” while 85% felt the timing of delivery was “perfect” (n=11/13).

None of the tailored text messages were used strictly as a reminder. All of the tailored text messages incorporated an aspect of the cognitive behavior skills associated with that week’s session. For example, parents were encouraged to make a plan and set SMART (specific, measurable, attainable, realistic, and timely) goals. After learning about that skill, a participant used the SMS to trigger physical activity with the message, “[Parent name]- Make a plan; schedule a SMART goal for family fun exercise; Remember- set yourself UP for SUCCESS! Believe you can do anything!” This message was delivered twice in one week, during the afternoon hours when the parent could make a plan and act on it. As parents advanced through the program, it appeared that they became more aware of how SMS content, timing, and frequency could cue them to act (or highlight an inability to act). It also became apparent that participants liked having a reminder of content covered in the face-to-face visit and corresponding section of the manual/audio CD to help them work on skills. For instance, one participant reported having the television on when her son was home in the afternoon, but wanted to work on increasing physical activity. She developed a combination text message with a reminder of where in the manual she could find help (Chapters 5 and 6), but also a tip on how she could build in a skill to meet her goal. At 4:30 pm, on a specified day, her text message was sent stating, “You're near the finish line. This wk 5&6! Decrease TV time or family exercise challenge on commercials! Who will win?”

### Static Text Messages

Parents reported not responding to the static text messages because they “thought it was a reminder” or “didn’t know to respond back.” The initial response rate was only 26% (15/58) for the static text messages. Subsequently, verbiage of the static text messages was made shorter and simpler, while topic and response algorithms remained unchanged. The response rate improved to 80% (38/47) with this change, ranging from 0%-100% response per participant (mean 49%, SD 36.2%). Overall, 87% of parents reported offering their child vegetables at dinner. Messages were confirmed delivered. The sociodemographic variability of the small sample was a factor in two-way SMS. At least two families had very limited SMS data plans. As a result, they could continue to receive text messages, but could not send them. Therefore, one participant did not respond to any of the static text messages (yes/no questions). Another parent circumvented her data plan by using a free software application to send outgoing text messages. While the service was free, it altered every outgoing message with a tag line advertising their service (eg, “Y -Sent free from TextNow.com”). This tag line was not part of the TEXT2COPE if/then algorithm, and subsequently fell into an aberrant response category. As a result, that participant did not receive immediate automated feedback to her response.

## Discussion

### Principal Findings

The results of this study indicate that a cognitive behavioral skills program, synergized with mobile messaging, is feasible and acceptable in a primary care setting with parents of OW/OB preschool-aged children. The preliminary short-term effects of this research show great potential for promoting healthy lifestyle choices in this population. There are a number of key points to be learned from this research. Using recommendations from Bowen [40], feasibility for this study was framed in several focus areas: (a) acceptability, (b) demand, (c) implementation, (d) practicality, (e) adaptation, and (f) integration.

### Acceptability

Parents reported high satisfaction with the TEXT2COPE program and expressed intent to continue using the skills learned in the program. The skills covered in the intervention align with previous research and perhaps shed light on retention and acceptance rates as well as preliminary effects. Better parental compliance with behavior strategies (eg, targeting specific behaviors, self-monitoring, goal setting, stimulus control/environmental cues, positive parenting strategies) is predictive of better outcomes for the child [7]. One parent commented that she copied (ie, cut and paste) the content from her TEXT2COPE text messages and programmed them into her electronic calendar so that her messages would continue to trigger the skills she wanted to practice. While the face-to-face visits allowed for detailed discussion on how to problem solve and set realistic goals, text messaging allowed for brief, real-time snippets of support. Parents desired a *strong message* that was *short* and *easy to read*. These findings parallel aspects of Fogg’s work suggesting that simplicity changes behavior [28], and effective behavior change is possible through smaller, habit-changing steps [41].

The use of static text messages warrants further exploration. The static text messages performed a multitude of functions. First, they served as a data collection tool with a varied response rate. The automated response built from the if/then algorithm served as reinforcement. Some parents viewed the text messages as reminders and did not respond. It is difficult to assess if response rate was a function of (a) the change in verbiage, (b) the way it was presented, or (c) characteristics of the participants responding. Predetermined delivery timing and vernacular could have contributed to the variability in response.

### Demand

Demand for innovative programs in the primary care setting reflects two domains: demand from the organization and demand from the patients. Office managers, providers, and staff welcomed the TEXT2COPE program as an innovative resource to treat OW/OB. However, partnering with primary care clinics was difficult. Administrative affiliations warranted lengthy negotiations between the PI and the organizations and required academic institutional review board approval, legal review, and/or third party reviewers. Parents stated that they welcomed the SMS communication from their providers. However, outside of the TEXT2COPE program, we were unaware of any direct communication between clinic and patients, outside of traditional phone calls or appointments. One clinic had an established Facebook page, yet, the clinic was unaware of how many families subscribed to their page or received updates. Parents also voiced an interest in learning more about how to help their child, but often lacked the resources. Parents reported feeling more proactive in their family’s health while using clinic time to address skills that could promote behavior change. Supplementing these new skills with text messaging provided additional support for parents in their home environment.

### Implementation

The intervention was manualized to facilitate reproducibility. A health care provider or lifestyle coach could deliver the content. With regards to SMS, sending weekly text messages took less than 5 minutes per participant, per text message. Automating text message delivery using machine learning techniques or learned user preferences is possible with advances in mHealth. From a logistical perspective, the use of text messages may require additional software, which would be either independent or programmed into existing electronic health record (EHR) software.

### Practicality

Practicality was explored when resources, existing means, and time were constrained in some way. Given the nonacute nature of healthy lifestyle behaviors, parents often requested to reschedule appointments. Parents often reported that some habits were more difficult to change than others. Mothers commonly reported that positive self-talk and “believing is achieving” was not something they commonly practiced; however, after reviewing this content more, mothers appreciated how this could affect the way serve as a role model to their children. Equally as impressive was that mothers welcomed content about topics such as overcoming barriers, problem solving, and goal setting. Positive reactions indicated parents favored an emphasis on “*how to* be healthy” versus being told, “you need to be healthier.” Parents were able to carry out those activities that were relevant to their needs. Congruent with the theoretical framework and previous research [8], this appeared crucial to participant interest.

### Adaptation

The user determined the number of weeks needed for content delivery, timing, and frequency of text message delivery. Most reflected parents’ routines around primary care use and their daily demands. However, adapting the schedule, content, and utilization of parent-driven text messages enhanced relevance to the user. Taveras and colleagues [42] reported that African American, Hispanic/Latino, and Asian parents rated the quality of nutrition and physical guidance received from health care professionals as poor to fair. Yet, cultural sensitivity is important to factor into the design of programs targeting healthy beliefs and behaviors. Therefore, content was designed and reinforced to incorporate the parent’s own words and build on parental understanding and use of the skills. The content manual provided a basis for knowledge about healthy lifestyle behaviors, while prior knowledge was incorporated into the face-to-face sessions as a starting point for discussion. For the pilot study, the manual was offered in print and audio format; future studies could offer a Web-based version of the manualized content for added flexibility.

### Integration

In terms of perceived fit, perceived sustainability, and costs to organizations and policies [40], the program was robust. The PI for this study also served as the interventionist, but was not a health care provider at the clinic sites. However, providers, support staff, and lifestyle coaches have the potential to implement this intervention. Providers could work within practice limitations, delivering the intervention in a typical 30-minute outpatient visit, and still provide evidence-based care, analogous to findings from Lusk and Melnyk [43,44]. Tailored SMS software can be integrated into existing EHR platforms and sustained over time. With the implementation of the Affordable Care Act, organizations have the opportunity to share in Medicare savings created through demonstrated quality performance [45]. Integrating the TEXT2COPE program can facilitate: (1) getting timely care and information, (2) how well your providers communicate, (3) patient ratings of providers, (4) health promotion and education, (5) shared decision making, and (6) health status.

### Strengths and Limitations

#### Strengths

This research sheds light on programs aimed at families with OW/OB preschoolers in a primary care setting, which have been sparse in the literature to date. Using a dynamic (versus static) design, the research supported participants as they progressed toward their goals. Parents want specific, action-oriented advice to achieve goals rather than general information on healthy behaviors [8]. While the sample was small, it was ethnically varied. The program was also designed to build skills relevant to the user’s goals, one skill and one goal at a time. The combination of text messages and clinic visits allowed for enhanced support through both small bursts of contact and traditional face-to-face time. The face-to-face visits were helpful when more detailed discussion was warranted about, for example, how to do something such as incorporate exercise into their daily routine. The text messages provided nontraditional support during times when the health care provider could not be there. By incorporating SMS, another channel of communication was opened with their child’s health care provider, a figure whom parents perceived as having a voice of authority [8].

#### Limitations

Although novel in approach, the findings must be tempered due to some limitations. First, the lack of an attention control group threatened the internal validity. Secondly, although fathers were encouraged to attend, the small sample size of only mothers (roughly 47% reported being single) limits the generalizability of the study findings. Perhaps a larger sample size and varying recruitment methods targeting dad’s groups could help to strengthen participation. Thirdly, measures were self-reported, wherein bias or human error can be introduced. Parent anthropometric measures would have been useful to obtain, as evidence suggests that one of the strongest predictors of childhood obesity is parental obesity [46]. Child anthropometric measures were collected at baseline; however, it may have been difficult to assess anthropometric changes over such a short period and those identified may not have accurately reflected the skills taught and changes observed in behavior. A longitudinal design incorporating parent and child anthropometric changes over time would be beneficial in future research.

### Conclusion

Mobile technologies, in conjunction with clinical care, have the potential to expand care and reduce costs. This new delivery method is poised to reinvent health care delivery, as it is superior to other technologies due to high demographic penetration, an omnipresent nature, fluidity of use, and a broad range of capabilities [47,48]. Feedback messages may play a pivotal role in goal attainment, through behavioral principles such as reinforcement and correction, and serve as a compass enabling individuals to stay on course [49]. However, there is strong potential for discord between patient/family desire for mHealth services and actual use in pediatric populations due to the scarcity of pediatric mHealth programs and evidence to successfully support clinical practice.

Future research should involve a full-scale randomized controlled trial to determine the short- and long-term efficacy of this intervention with families who have OW/OB preschool children. Technology also lends itself to alternate research methods such as adaptive interventions. Highly adaptive sequential multiple assignment randomized trials (SMART) have been used to inform the best sequencing of treatments when individuals are not responding, and the best time to transition from more to less intensive or maintenance therapy [50-53]. Adaptive interventions may be ideal for families attempting to change behavior amid already hectic schedules. *Crunch time* (3:00 pm to bedtime during the work week) was found to be a major contributor to unhealthy habits for millions of American children [54]. While 95% of parents polled believed that it is important for their kids to eat healthy and exercise, more than half of the parents (60%) said their children ate or drank something unhealthy during *crunch time*, did not get enough physical activity, and reported eating out 6 or 7 nights in the past week (48%) [54]. Similarly, in the TEXT2COPE study, most tailored text messages were requested to trigger a response in the late afternoon hours. Perhaps SMS delivery reinforced acceptability of the program by providing participants with support during times when support was needed the most. Previous research demonstrates that interventions may be more effective if the process of behavior change is emphasized, rather than the health-related outcomes [55]. In this study, during the face-to-face sessions and in subsequent messaging, some parents focused on the actual habit (ie, exercise), while some focused on the actual skill (ie, problem-solving, goal-setting). It would be interesting to further test the specific effects of cognitive versus behavioral training on healthy lifestyle related outcomes. With continued efforts, proof of concept and proof of efficacy studies will provide insight into mHealth strategies used in the field of pediatrics.

## Abbreviations

CBT: cognitive behavioral therapy
CT: Cognitive Theory
FBM: Fogg’s Behavior Model
OW/OB: overweight/obese
SMS: short message service
